# Energy drinks: effects on pediatric 24-h ambulatory blood pressure monitoring. A randomized trial

**DOI:** 10.1038/s41390-023-02598-y

**Published:** 2023-04-15

**Authors:** Felix S. Oberhoffer, Robert Dalla-Pozza, André Jakob, Nikolaus A. Haas, Guido Mandilaras, Pengzhu Li

**Affiliations:** Division of Pediatric Cardiology and Intensive Care, University Hospital, LMU Munich, 81377 Munich, Germany

## Abstract

**Background:**

Energy drinks (EDs) are popular beverages among minors. To date, clinical studies investigating ED-induced effects on the pediatric cardiovascular system are sparse. This study aimed to investigate the effects of a single, bodyweight-adjusted ED dosage on 24-h ambulatory blood pressure monitoring (ABPM) in healthy children and adolescents.

**Methods:**

This study was a randomized, single-blind, placebo-controlled, crossover clinical trial. Study participants received a single, bodyweight-adjusted ED amount or a placebo drink on 2 consecutive days at similar morning hours. Twenty-four-hour ABPM was assessed via an automated oscillometric blood pressure device after beverage consumption on both study days.

**Results:**

A total of 17 healthy children and teenagers (13.90 (12.29–17.89) years) were included in the final analysis. The ED consumption led, compared to the placebo intake, to a significantly higher 24-h systolic (115.90 (110.22–118.04) vs. 110.64 (108.09–115.45) mmHg, *p* = 0.013) and diastolic blood pressure (66.08 (64.20–68.32) vs. 62.63 (61.40–66.46) mmHg, *p* = 0.005).

**Conclusions:**

The single, bodyweight-adjusted ED consumption is linked with a significantly higher systolic as well as diastolic 24-h blood pressure in healthy children and adolescents. Minors, particularly those with an increased cardiovascular morbidity, should be discouraged from drinking EDs.

**Impact:**

- Energy drinks (EDs) are consumed by many children and teenagers. While adverse cardiovascular events after ED consumption were reported in the literature, the effects of these beverages on the pediatric 24-h blood pressure profile have not been systematically evaluated yet. In our manuscript, we demonstrate for the first time that acute ED consumption is associated with a significantly higher 24-h systolic blood pressure and diastolic blood pressure in healthy minors.

## Introduction

Energy drinks (EDs) are sweetened beverages that contain multiple stimulants such as caffeine, guarana, or taurine. Since their market introduction in the 1980s, EDs have experienced massive marketing.^1–3^ EDs are advertised as physical as well as mental performance enhancers and are particularly popular among the younger population; according to a survey conducted by the European Food Safety Authority (EFSA), 68% of adolescents reported drinking EDs.^1^ Among them, 26% drink EDs at least 2 days per week.^1^

Closely linked with their popularity, reports on adverse cardiovascular events, such as arterial hypertension or cardiomyopathy, associated with the consumption of EDs are rising.^4–7^ Even though children and teenagers are considered the main ED consumer group,^1^ clinical studies investigating ED-induced effects on the pediatric cardiovascular system are lacking.

A recent study conducted by our department evaluated the acute effects of ED consumption on clinic blood pressure in healthy children and adolescents.^8^ In this particular study, participants consumed a single, bodyweight-adjusted ED amount and a placebo beverage matched in sugar content but without conventional ED ingredients.^8^ Systolic (SBP, mmHg) and diastolic blood pressure (DBP, mmHg) were measured at baseline as well as 30, 60, 120, and 240 min after beverage consumption.^8^ Interestingly, mean clinic SBP was shown to be up to 5.23 mmHg (*p* < 0.0001) and mean clinic DBP up to 3.29 mmHg (*p* < 0.001) higher after the ED intake when compared to the placebo consumption.^8^

While these results indicate a significant ED-induced raise in clinic blood pressure within a 4-h period, it remains unclear whether acute ED consumption also affects the pediatric 24-h blood pressure profile. To the best of our knowledge, studies focusing on the ED-induced effects on 24-h ambulatory blood pressure monitoring (ABPM) are sparse and have not been conducted in a pediatric cohort yet.^9^ However, multiple studies suggest that 24-h blood pressure is a stronger predictor of all-cause mortality and cardiovascular mortality than clinic blood pressure.^10–13^ Moreover, the assessment of 24-h blood pressure can be regarded as crucial for additional and precise cardiovascular risk assessment.

Therefore, this study aimed to investigate the effects of a single, bodyweight-adjusted ED dosage on 24-h ABPM in healthy children and adolescents.

## Methods

### Study population

Detailed information on the study population and design was given in recent publications conducted by our department and will be shortly summarized in the following.^8,14–16^ For this study, healthy children and adolescents aged 10–18 years were prospectively enrolled within the greater Munich (Germany) area via public calls. Before enrollment, study participants were examined for eligibility. Exclusion criteria were as follows: presence of chronic conditions (e.g., congenital heart disease, arterial hypertension, presence of severe dysrhythmia), history of sudden heart death within the family, known allergies against beverage ingredients, regular use of medication as well as the use of pro re nata medication with effects on the cardiovascular function, regular use of drugs including smoking and alcohol consumption, pregnancy. Bodyweight (kg) and height (cm) were assessed. In minor study participants, weight classification was defined according to body mass index (BMI, kg/m^2^) percentiles (P.) for a German reference population.^17^ Weight classification in study participants ≥18 years of age was determined as follows: normal weight if BMI <25 kg/m^2^, overweight if BMI ≥25 kg/m^2^ but <30 kg/m^2^, obese if BMI ≥30 kg/m^2^. Caffeine consumption behavior was defined according to Shah et al.^18^ For the assessment of ED consumption behavior, similar definitions were applied.^8,14–16^

### Study design

This study was a randomized, single-blind (study participants), placebo-controlled, crossover clinical trial that was conducted between April 2021 and October 2021 by the Division of Pediatric Cardiology and Intensive Care, University Hospital, LMU Munich (Munich, Germany). The study was registered in the German Clinical Trials Register (https://www.drks.de/drks_web/, DRKS00027580).

Subjects were asked to abstain from caffeine (e.g., personal ED intake, coffee, tea, chocolate) and drugs (e.g., tobacco, alcohol) 48 h before and 24 h after study participation. Prior to each study day, an overnight fast (with allowance of water) was requested. Study participants were asked not to consume any food or liquids within the first 4 h after beverage consumption. The randomization into one of two intervention phases was executed by coin flipping. Study participants were given either a commercially available caffeinated ED or a placebo drink without the typical ED components (e.g., caffeine, taurine) on 2 consecutive days. The administered ED dosage was bodyweight-adjusted (3 mg caffeine per kilogram of bodyweight) and reflected the maximum daily caffeine intake for healthy children and adolescents as proposed by the EFSA.^19^ The provided amount of placebo was matched to that of the ED. Specific ingredients of both beverages were given in a recent publication conducted by our department.^8^ The ED contained caffeine (32 mg/100 mL), taurine (200 mg/100 mL), glucuronolactone (24 mg/10 mL), ginseng aroma extract (10 mg/100 mL), guarana extract (10 mg/100 mL) and vitamins. The placebo drink contained carbonated water, multi-fruit juice, fruit extracts, and vegetable extracts. ED and placebo drinks were similar in sugar content (ED: 15.2 g/100 mL, placebo drink: 13.2 g/100 mL) and taste. Both drinks were given at comparable morning hours in an indistinguishable and masked drinking bottle. To reduce the potential impact of physical activity on the cardiovascular parameters investigated in previous publications conducted by our department,^8,14–16^ study participants were admitted to our pediatric ward and given a sickbed for each study day. Study participants remained supine within a 4-h monitoring period after beverage consumption. After the 4-h monitoring period, subjects were discharged from our pediatric ward. To assess blinding quality, study participants were asked to guess the day of ED administration after completed data collection.

### Twenty-four-hour ambulatory blood pressure monitoring

To rule out potential baseline differences, clinic SBP and DBP were measured through an automated blood pressure device (Infinity M540, Dräger, Germany) on both study days before beverage consumption. For 24-h ABPM, an automated oscillometric blood pressure device (Mobil-O-Graph®, IEM, Germany) was utilized. Cuff sizes were selected according to the right upper arm circumference. The 24-h ABPM measurement was initiated simultaneously with beverage consumption. The device performed measurements automatically every 15 min during daytime and every 30 min during nighttime. Study participants were asked to note sleep and wake times on a separate protocol. The assessment of 24-h ABPM data was conducted by a masked researcher. Measurement errors were removed manually from the data set. Participants were included in the final analysis if for each study day ≥50 blood pressure measurements were successfully recorded within a 24-h period and if ≥10 blood pressure measurements were successfully performed during nighttime. Mean values of the following parameters were assessed for daytime, nighttime, and the total 24-h period: SBP, DBP, mean arterial pressure (MAP, mmHg), pulse pressure (PP, mmHg), central systolic blood pressure (cSBP, mmHg), central diastolic blood pressure (cDBP, mmHg), central pulse pressure (cPP, mmHg), augmentation index normalized for a heart rate of 75 bpm (AIx@75, %), pulse wave velocity (PWV, m/s), and heart rate (HR, bpm).

Compared to intra-aortic readings in adult patients, central blood pressure and PWV measurements assessed by the Mobil-O-Graph® were demonstrated to be accurate and acceptable.^20,21^

Dipping (%) was calculated for SBP and DBP individually as follows:

$${{{{{\mathrm{Dipping}}}}}}\,({{{{{{{\mathrm{\% }}}}}}}}) = \frac{{({{{{{\mathrm{Mean}}}}}}\;{{{{{\mathrm{Blood}}}}}}\;{{{{{\mathrm{Pressure}}}}}}\;{{{{{\mathrm{Daytime}}}}}} - {{{{{\mathrm{Mean}}}}}}\;{{{{{\mathrm{Blood}}}}}}\;{{{{{\mathrm{Pressure}}}}}}\;{{{{{\mathrm{Nightime}}}}}})}}{{{{{{{\mathrm{Mean}}}}}}\;{{{{{\mathrm{Blood}}}}}}\;{{{{{\mathrm{Pressure}}}}}}\;{{{{{\mathrm{Daytime}}}}}}}} \times 100.$$

In addition, the percentage (%) of SBP and DBP measurements classified as arterial hypertension was calculated for daytime, nighttime, and the total 24-h period as follows: in study participants <16 years of age, arterial hypertension at daytime was present if blood pressure was ≥95 P. of a pediatric reference population.^22,23^ In study subjects ≥16 years of age, arterial hypertension was present if blood pressure was ≥130/80 mmHg during daytime.^24^ At nighttime, arterial hypertension was present if blood pressure was ≥90% of the above-mentioned daytime cut-offs.

### Assessment of sleep duration and quality

Study participants were asked to note sleep- and wake times on a separate protocol allowing the calculation of sleep duration for each study day. Moreover, participants were asked to rate sleep quality using an unvalidated “smiley” scale (1 = very bad, 2 = bad, 3 = average, 4 = good, 5 = very good) for each study day.

### Endpoint measurements

The following vascular endpoints assessed for daytime, nighttime, and the total 24-h period were included in the analysis: SBP, DBP, MAP, PP, cSBP, cDBP, cPP, AIx@75, PWV, and HR. Additional endpoints considered for analysis were: SBP and DBP dipping, percentage of SBP and DBP measurements classified as arterial hypertension, sleep duration, sleep time, wake time, and sleep quality. All endpoints were measured on each study day.

### Statistical analysis

This study was a pediatric pilot study. To the best of our knowledge, pediatric reference values regarding the change in 24-h ABPM after ED consumption do not exist and, thus, could not be considered in a power analysis. However, in the literature, a study conducted by Franks et al. displayed a significantly higher mean 24-h SBP and DBP in nine healthy adult subjects after receiving an ED beverage at multiple time points, compared to a caffeinated placebo drink.^9^ Hence, for this pediatric pilot study, a sample size ≥9 was aimed. To test for normal distribution of continuous variables, the Shapiro–Wilk test, histograms, and QQ plots were used. To compare 24-h ABPM data between ED and placebo group, a paired *t*-test was applied for normally distributed data, and a Wilcoxon-signed-rank test was applied for non-normally distributed data. Data analysis was independently performed by a masked researcher using SPSS (IBM SPSS Statistics 27.0). A *p* value <0.05 was considered statistically significant.

## Results

### Study participants’ characteristics

A total of 27 children and adolescents were recruited for the present study. Due to insufficient data quality of 24-h ABPM, 10 study participants had to be excluded from further analysis. In total, 17 study participants were included in the final analysis.

None of the study participants displayed pre-existing health conditions nor reported the use of medication. Seven out of 17 study participants (41.18%) correctly guessed the day of ED administration, suggesting appropriate blinding quality. There was no significant difference in age between participants who correctly guessed the day of ED administration and those who did not (14.49 ± 2.37 vs. 14.91 ± 2.76 years, *p* = 0.75).

Clinic SBP (112.53 ± 8.09 vs. 111.29 ± 7.78 mmHg, *p* = 0.98) and DBP (64.35 ± 8.46 vs. 64.24 ± 6.61 mmHg, *p* = 0.95) did not show significant differences between ED and placebo drink at baseline.

Table 1 summarizes information on study participants’ characteristics.

**Table 1:** Study participants’ characteristics (*n* = 17).

Characteristics	Total
Age (years)	13.90 (12.29–17.89)
Sex, *n* (%)	
Male	9 (52.94)
Female	8 (47.06)
Bodyweight (kg)	53.44 ± 12.00
Height (cm)	166.28 ± 13.19
Weight classification, *n* (%)	
Normal weight	15 (88.24)
Overweight	2 (11.76)
Obese	0 (0)
Caffeine consumption behavior, *n* (%)^a^	
Rarely	10 (58.82)
Occasionally	2 (11.76)
Frequently	3 (17.65)
Daily	2 (11.76)
Energy drink consumption behavior, *n* (%)^b^	
Never	7 (41.18)
Rarely	8 (47.06)
Occasionally	1 (5.88)
Frequently	1 (5.88)
Daily	0 (0)

Continuous data are given as median (IQR) if non-normally distributed. Nominal data are presented as *n* (%).

^a^Rare caffeine consumers if <1 caffeine-containing drink per month, occasional caffeine consumers if 1–3 caffeine-containing drinks per month, frequent caffeine consumers if 1–6 caffeine-containing drinks per week, and daily caffeine consumers if ≥1 caffeine-containing drink per day.^18^

^b^Rare energy drink consumers if <1 energy drink per month, occasional energy drink consumers if 1–3 energy drinks per month, frequent energy drink consumers if 1–6 energy drinks per week, and daily energy drink consumers if ≥1 energy drink per day.

### Effects of energy drinks on 24-h ambulatory blood pressure measurement

The single, bodyweight-adjusted ED consumption during the morning hours led to a significantly higher SBP, DBP, MAP, cSBP, cDBP, and a significantly higher prevalence of DBP measurements classified as arterial hypertension at daytime compared with the placebo beverage. On average, SBP, DBP, and MAP at daytime were 3.33%, 4.50%, and 3.86% higher after ED consumption.

In addition, DBP and cDBP at nighttime displayed significantly higher values after acute ED consumption. On average, SBP, DBP, and MAP at nighttime were 2.88%, 3.74%, and 3.18% higher after ED consumption.

Within the 24-h period after beverage consumption, a significantly higher SBP, DBP, MAP, cSBP, and cDBP was observed in the ED group. Twenty-four-hour PWV tended to be higher after the acute ED consumption but did not reach statistical significance. On average, 24-h SBP, DBP, and MAP were 3.33%, 4.76%, and 3.92% higher after ED consumption.

**Fig. 1:**
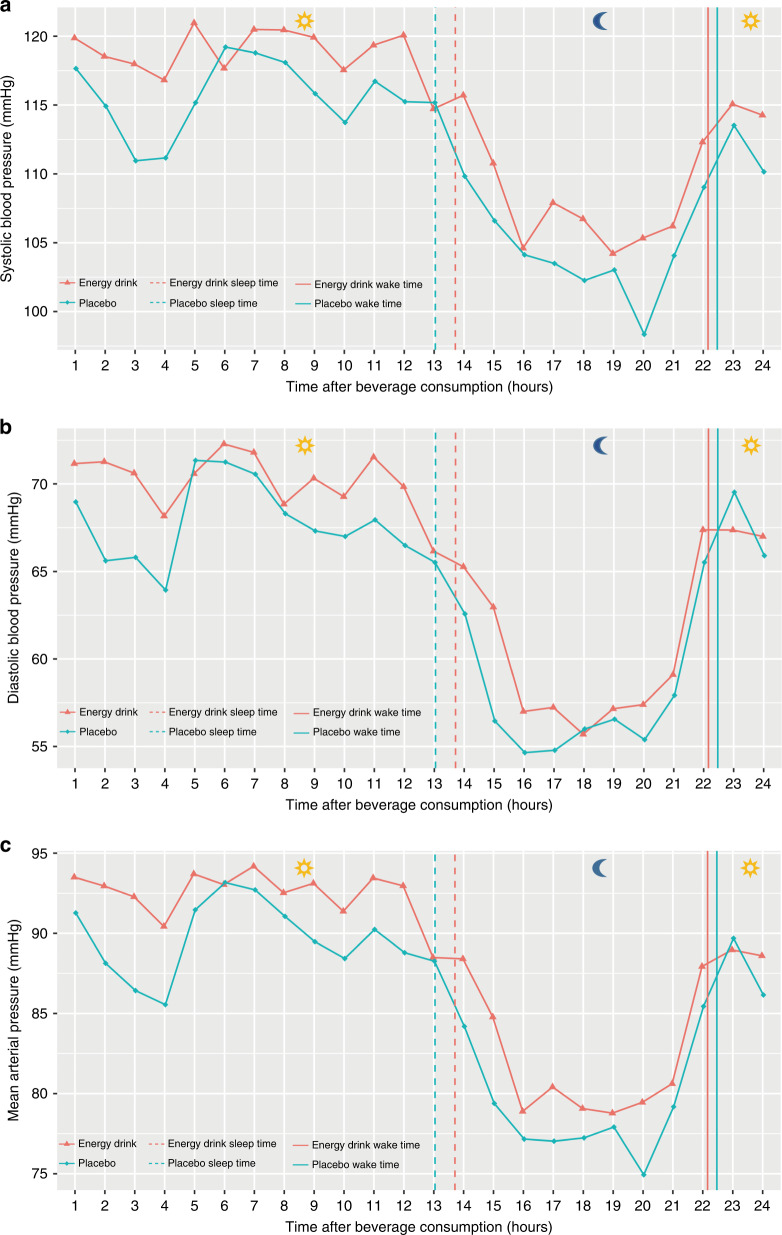
Twenty-four-hour blood pressure profile after energy drink and placebo beverage consumption (*n* = 17). Systolic blood pressure (**a**), diastolic blood pressure (**b**), mean arterial pressure (**c**). Beverages were consumed at similar morning hours (Time 0). An automated oscillometric blood pressure device performed measurements every 15 min during daytime and every 30 min during nighttime. Study participants were asked to note sleep and wake time on a separate protocol.

Table 2 gives detailed information on 24-h ABPM for daytime, nighttime, and the overall 24-h period after beverage consumption. Moreover, Fig. 1 demonstrates average SBP, DBP, and MAP profile during the 24-h period after ED and placebo beverage consumption.

**Table 2:** Effects of energy drinks on 24-h ambulatory blood pressure measurement (*n* = 17).

	Daytime			Nighttime			24-h period		
	Energy drink	Placebo	*p* value	Energy drink	Placebo	*p* value	Energy drink	Placebo	*p* value
ABPM measurements (*n*)	50.76 ± 10.29	47.94 ± 12.34	0.300	17.00 (15.50–18.50)	19.00 (16.00–22.00)	0.144	67.88 ± 9.34	67.29 ± 9.93	0.814
ABPM duration (h)	13.78 ± 1.32	13.10 ± 2.05	0.248	7.55 ± 0.92	8.43 ± 1.70	0.041*	22.28 ± 1.18	22.08 ± 1.33	0.626
SBP (mmHg)	118.02 (113.76–121.89)	115.53 (109.36–120.19)	0.025*	106.65 (99.38–112.07)	103.05 (98.01–107.79)	0.163	115.90 (110.22–118.04)	110.64 (108.09–115.45)	0.013*
Hypertensive SBP measurements (%)	17.86 (8.55–30.26)	11.67 (1.74–23.94)	0.068	20.00 (8.20–38.89)	9.09 (3.56–20.45)	0.098	16.67 (7.69–36.72)	11.11 (3.60–23.45)	0.055
SBP dipping (%)	–			10.19 ± 4.59	9.65 ± 5.19	0.743	–		
DBP (mmHg)	69.07 (67.25–71.71)	66.35 (64.16–69.51)	0.003**	58.50 ± 5.39	56.41 ± 3.77	0.047*	66.08 (64.20–68.32)	62.63 (61.40–66.46)	0.005**
Hypertensive DBP measurements (%)	10.71 (8.09–26.14)	10.64 (4.17–16.89)	0.049*	0 (0–6.25)	2.78 (0–8.01)	0.875	8.43 (6.90–19.90)	8.54 (3.18–13.64)	0.062
DBP dipping (%)	–			15.99 (13.86–20.58)	14.13 (11.75–19.75)	0.554	–		
MAP (mmHg)	90.82 (89.42–94.41)	88.56 (85.17–91.99)	0.006**	80.55 ± 6.57	78.15 ± 5.49	0.073	89.75 (86.08–90.88)	85.12 (83.09–88.02)	0.006**
PP (mmHg)	48.46 ± 6.62	47.72 ± 6.39	0.362	47.71 (44.39–51.94)	45.88 (42.45–50.13)	0.554	48.40 ± 6.17	47.70 ± 6.16	0.368
cSBP (mmHg)^a^	103.70 ± 6.05	101.33 ± 8.19	0.043*	96.86 ± 7.71	93.79 ± 7.90	0.099	101.77 ± 5.64	99.18 ± 7.93	0.019*
cDBP (mmHg)^a^	71.50 (69.77–73.99)	68.75 (66.75–70.78)	0.004**	59.69 ± 5.65	57.19 ± 3.82	0.047*	68.47 (65.71–70.37)	63.99 (63.10–67.61)	0.008**
cPP (mmHg)^a^	30.93 ± 5.22	31.57 ± 5.16	0.335	37.13 ± 4.68	36.62 ± 5.24	0.635	32.71 ± 4.68	33.12 ± 4.72	0.471
AIx@75 (%)^a^	17.23 ± 5.65	16.50 ± 5.50	0.489	8.84 ± 3.77	9.29 ± 6.62	0.800	14.83 ± 4.77	14.21 ± 5.40	0.595
PWV (m/s)^a^	4.70 (4.59–4.83)	4.67 (4.46–4.89)	0.255	4.49 ± 0.34	4.37 ± 0.31	0.100	4.63 (4.54–4.74)	4.58 (4.42–4.69)	0.056
HR (bpm)^a^	74.78 ± 8.98	75.87 ± 8.15	0.356	60.90 ± 6.81	61.53 ± 8.99	0.649	70.78 (65.14–76.20)	70.64 (66.47–76.61)	0.586

Data are presented as mean ± SD if normally distributed and as median (IQR) if non-normally distributed.

*ABPM* ambulatory blood pressure measurement, *SBP* systolic blood pressure, *DBP* diastolic blood pressure, *MAP* mean arterial pressure, *PP* pulse pressure, *cSBP* central systolic blood pressure, *cDBP* central diastolic blood pressure, *cPP* central pulse pressure, *AIx@75* augmentation index normalized for a heart rate of 75 bpm, *PWV* pulse wave velocity, *HR* heart rate.

*p value <0.05; **p value <0.01.

^a^16 study participants were included in the analysis.

### Effects of energy drinks on sleep duration and quality

**Fig. 2:**
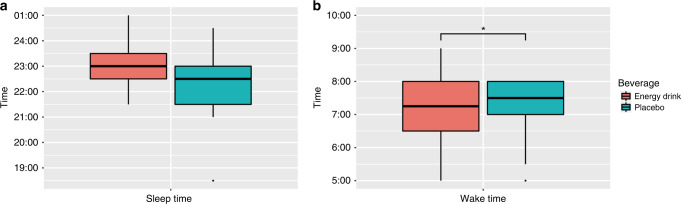
Sleep time and wake time after energy drink and placebo beverage consumption (*n* = 17). Sleep time (**a**), wake time (**b**), *p <0.05.

**Fig. 3:**
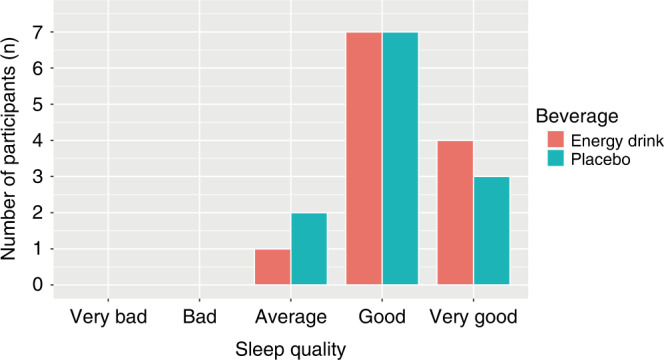
Sleep quality after energy drink and placebo beverage consumption (*n* = 12). Five out of 17 study participants gave incomplete information on sleep quality and were excluded from the analysis.

The duration of sleep was significantly shorter after the acute ED consumption (7.55 ± 0.92 vs. 8.43 ± 1.70 h, *p* = 0.041). Sleep time did not differ significantly between ED and placebo groups (23:00 (22:00–23:30) vs. 23:00 (21:30–23:00), *p* = 0.304) (Fig. 2). Wake time was significantly earlier in the ED group compared to the placebo group (7:00 (6:30–7:30) vs. 8:00 (7:00–8:00), *p* = 0.027) (Fig. 2). Sleep quality was self-reported by participants ranging from very good to very bad. Five out of 17 study participants gave incomplete information on sleep quality and were excluded from the analysis. No significant difference was found concerning sleep quality after ED and placebo beverage consumption (Fig. 3).

## Discussion

To the best of our knowledge, this is the first study investigating the acute effects of ED consumption on 24-h ABPM in healthy children and adolescents. In total, 17 study participants were included in the final analysis. The results of this study revealed a significantly altered 24-h ambulatory blood pressure profile, including a significantly higher 24-h SBP and DBP, after the single, bodyweight-adjusted ED consumption. In addition, 24-h PWV, a marker of arterial stiffness, tended to be higher after acute ED consumption.

EDs contain multiple ingredients. In the following, their potential effects on the pediatric cardiovascular system will be elaborated further. High amounts of caffeine (32 mg/100 mL) and its derivates (guarana extract, 10 mg/100 mL) were present in the ED used for this study.^8^ Caffeine is thought to promote left ventricular inotropy, act vasoconstrictive, and therefore raise blood pressure.^25^ Studies on adult subjects demonstrated that left ventricular contractility was significantly elevated after acute ED consumption.^26,27^ Recently, our department was able to show similar results for pediatric subjects.^16^ Moreover, a recent publication conducted by our department revealed that acute ED consumption was linked with a significantly increased arterial stiffness of the common carotid arteries in healthy children and adolescents.^15^ The current study displayed a higher tendency of 24-h PWV, an additional marker of arterial stiffness, after acute ED consumption, reinforcing the above-mentioned findings. As reported in the introduction, a previous publication conducted by our department indicated a significant ED-induced raise in clinic blood pressure within a 4-h period.^8^ Interestingly, the single, bodyweight-adjusted ED consumption during the morning hours led to a significantly higher SBP, DBP, MAP, cSBP, cDBP and a significantly higher prevalence of DBP measurements classified as arterial hypertension during daytime compared with the placebo beverage. Within the 24-h period after beverage consumption, significantly higher SBP, DBP, MAP, cSBP, and cDBP were observed in the ED group.

The results presented in this study are in line with the findings by Franks et al.^9^ The authors investigated the effects of EDs versus caffeine supplementation on 24-h ABPM in nine healthy adults.^9^ Subjects received an ED (80 mg caffeine) or a placebo (80 mg caffeine) at four time points throughout the day (08, 11, 15, and 19 h).^9^ The authors revealed a 5.8 mmHg higher mean 24-h SBP (*p* = 0.04) and a 5.4 mmHg higher mean 24-h DBP (*p* = 0.02) on the day of ED consumption.^9^ It should be noted that both study designs are not fully comparable, as the current study used a non-caffeinated placebo drink, and both beverages were given only once at similar morning hours. However, the study of Franks et al. suggests that the various ingredients found in EDs may potentiate blood pressure response to caffeine due to pharmacodynamic or pharmacokinetic interactions.^9^ In the literature, caffeine plasma half-lives are described to range between 2.5 and 5 h, if administered in a single dose of 4 mg per kilogram of bodyweight.^28^ Moreover, with half-lives ranging from 2.3 to 9.9 h, caffeine clearance can exhibit considerable interindividual variability.^28^ The results of the current study could potentially imply that the pediatric cardiovascular system reacts more sensitively to ED ingredients than the adult one. Furthermore, the ED-induced effects on the pediatric cardiovascular system might last distinctly longer.

The duration of sleep was significantly shorter after the acute ED consumption. However, sleep quality did not differ significantly between both beverages. As caffeine increases cortisol secretion, it is proposed to influence the hypothalamic–pituitary–adrenal axis and, thus, the sleep-wake cycle.^29^ Multiple studies indicate that the consumption of caffeine is associated with shorter sleep duration as well as elevated sleep onset latency and wake time after sleep onset.^30^ A study conducted by Drake et al. demonstrated that the consumption of 400 mg caffeine 6 h prior to habitual sleep time resulted in a significant reduction of sleep duration in 12 healthy normal sleepers.^31^ Therefore, the consumption of large caffeine amounts, even at daytime, can negatively affect sleep.^31^ Furthermore, a survey conducted by Sampasa-Kanyinga et al. suggests that the consumption of EDs is linked with a shorter sleep duration in middle as well as in high school students.^32^

Regarding the 24-h blood pressure profile of study participants in this study, the significantly shorter duration of sleep after the ED intake, potentially due to a caffeine-induced increase of cortisol secretion,^29^ might have substantially contributed to the demonstrated blood pressure rise. Nighttime DBP and cDBP displayed significantly higher values after acute ED consumption. The remaining vascular parameters at nighttime, including SBP and DBP dipping, did not show significant differences between both beverages. In adolescents aged 13–18 years, a sleep duration between 8 and 10 h per 24 h is recommended for optimal health promotion.^33^ Excessively shorter sleep duration is linked with arterial hypertension.^34^ Cappuccio et al. revealed that a short sleep duration is associated with a higher risk of developing or dying of coronary heart disease and stroke.^35^

In addition to caffeine, EDs often include taurine, glucuronolactone, and B vitamins. While the aminoacidic taurine is thought to decrease blood pressure,^36,37^ further research is needed to determine the effects of glucuronolactone and B vitamins on the cardiovascular system.

The results of this study reveal that the consumption of a single, bodyweight-adjusted ED dosage is linked with a significantly higher median 24-h SBP (+5.26 mmHg) and DBP (+3.45 mmHg), compared to a placebo beverage, in healthy children and adolescents. A meta-analysis by Conen et al. suggests that an increase of 10 mmHg in 24-h SBP is connected with a 27% higher risk for cardiovascular events.^38^ In addition, a population-based study by Hansen et al. indicates that an increase of 5 mmHg in 24-h DBP is associated with a 27% higher risk for cardiovascular disease.^39^ Therefore, the ED-induced alterations in the pediatric 24-h blood pressure profile displayed in this study can be considered alarming.

Moreover, chronic ED consumption could result in arterial hypertension and hence increased left ventricular afterload, ultimately leading to left ventricular dysfunction and hypertrophy.^16^ A previous publication conducted by our department reported a significantly lower cardiac efficiency after acute ED consumption in healthy children and adolescents.^16^ Furthermore, many EDs contain high amounts of sugar and thus “empty” calories. Chronic ED consumption could therefore lead to the onset of glucose metabolism disorders and aggravate weight gain. As ED consumption is associated with a shorter sleep duration,^32^ it might additionally increase cardiovascular risk.^35^ In summary, children and adolescents, particularly those with elevated cardiovascular morbidity (e.g., arterial hypertension, diabetes, excess weight), should be discouraged from consuming EDs. Moreover, minors should be made aware of the potential health risks of excessive ED intake as well as responsible ED consumption behaviors.

As a survey conducted by the EFSA suggests, ED consumption is very popular among children and adolescents, which could pose potential public health concerns considering the demonstrated results of this study.^1^ In total, 68% of adolescents stated to drink EDs.^1^ Of those, 26% reported drinking EDs at least 2 days per week.^1^ Medical organizations such as the American Academy of Pediatrics advise against the use of EDs in minors.^40^ Due to negative health concerns, some countries, including Lithuania and Latvia, have already prohibited selling EDs to minors.^41,42^

The results of the EDUCATE-Study (Energy-Drinks—Unexplored Cardiovascular Alterations in TEens and TwEens) can be regarded as noteworthy since they offer policymakers with long-needed data on the cardiovascular effects of acute ED consumption in children and adolescents.

Strengths and limitations of the EDUCATE-Study have already been covered in previous publications conducted by our department and will be shortly summarized in the following.^8,14–16^ To the best of our knowledge, this is the first pediatric, and the largest study ever, examining the effects of a single, bodyweight-adjusted ED dosage on 24-h ABPM. Still, the sample size of this pilot study can be regarded as relatively low. The vascular parameters were assessed automatically by an oscillometric blood pressure device, and data analysis was independently performed by a masked researcher. However, some bias may have resulted due to the single-blind (study participants) study design. Adequate blinding quality can be suggested as only 41.18% of study participants accurately guessed the day of ED administration. Even though both beverages were similar in taste and were given in an indistinguishable and masked drinking bottle, some study participants might have recognized the given drink by taste, smell, or physical response. Moreover, the assessment of sleep duration and quality was based on subjective recalls by study participants and not on objective measures. For the assessment of sleep quality, a non-validated questionnaire was used. Due to ethical as well as safety reasons, the ED amount was matched according to the maximum daily caffeine intake for healthy children and adolescents (3 mg caffeine per kilogram of bodyweight) as proposed by the EFSA.^19^ The EFSA justifies this cut-off as caffeine clearance in minors is at least that of adults.^19^ Interestingly, mean acute exposure to caffeine from ED under adolescent ED consumers is 2.92 mg per kilogram of bodyweight as a survey conducted by the EFSA suggests.^1^ Therefore, we consider the current study to reflect realistic ED consumption behaviors under minors. However, only one ED product was used in this study. Moreover, the pediatric circulatory system may respond differently to greater ED amounts, other ED products, the concomitant use of EDs with drugs (e.g., alcohol), or in the presence of an increased cardiovascular risk (e.g., arterial hypertension, diabetes, excess weight). Hence, future research on this matter is required. For a more precise classification of study participants’ cardiovascular risk, future studies should not only use conventional variables such as BMI but also body fat percentage as well as lean body mass. Subjects at different developmental stages were included in this study. However, the cardiovascular response to EDs potentially differed depending on each individual’s baseline hormone level.^43^ The vascular parameters studied were potentially influenced by habitual caffeine effects as well as interindividual caffeine responses.^8,44^ As the current study design did not require a washout period of numerous days between the consumption of both beverages, study participants who received the ED on the first study day might not have fully eliminated caffeine from their system prior to consuming the placebo drink.^28^ In this study, salt intake was not controlled the night before and during data collection. Moreover, diet and physical activity diaries were not kept by study participants. Lastly, the chronic effects of EDs on blood pressure remain unclear and require further investigation.

## Conclusion

The single, bodyweight-adjusted ED consumption during morning hours is linked with a significantly higher systolic as well as diastolic 24-h blood pressure in healthy children and adolescents. Potentially, chronic ED consumption might lead to greater cardiovascular risk. Therefore, further studies are required to investigate the chronic effects of EDs on the cardiovascular system. Minors, particularly those with an increased cardiovascular morbidity, should be discouraged from drinking EDs.

## Data Availability

The data presented in this study are available upon reasonable request from the corresponding author.
